# Specialized nutrition improves muscle function and physical activity without affecting chemotherapy efficacy in C26 tumour‐bearing mice

**DOI:** 10.1002/jcsm.12703

**Published:** 2021-05-06

**Authors:** Liza A. Wijler, Danielle A.E. Raats, Sjoerd G. Elias, Francina J. Dijk, Hanil Quirindongo, Anne M. May, Matthew J.W. Furber, Bram Dorresteijn, Miriam van Dijk, Onno Kranenburg

**Affiliations:** ^1^ Laboratory of Translational Oncology, Division of Imaging and Cancer University Medical Centre Utrecht, Utrecht University Utrecht The Netherlands; ^2^ Department of Epidemiology, Julius Center for Health Sciences and Primary Care University Medical Centre Utrecht, Utrecht University Utrecht The Netherlands; ^3^ Danone Nutricia Research Utrecht The Netherlands; ^4^ Utrecht Platform for Organoid Technology Utrecht University Utrecht The Netherlands

**Keywords:** Colorectal cancer, Specialized nutrition, Muscle function, Pre‐cachexia, Physical activity

## Abstract

**Background:**

Skeletal muscle wasting and fatigue are commonly observed in cancer patients receiving chemotherapy and associated with reduced treatment outcome and quality of life. Nutritional support may mitigate these side effects, but potential interference with chemotherapy efficacy could be of concern. Here, we investigated the effects of an ω‐3 polyunsaturated fatty acid (eicosapentaenoic acid and docosahexaenoic acid), leucine‐enriched, high‐protein (100% whey), additional vitamin D, and prebiotic fibres ‘specific nutritional composition’ (SNC) and chemotherapy on state‐of‐the‐art tumour organoids and muscle cells and studied muscle function, physical activity, systemic inflammation, and chemotherapy efficacy in a mouse model of aggressive colorectal cancer (CRC).

**Methods:**

Tumour‐bearing mice received a diet with or without SNC. Chemotherapy treatment consisted of oxaliplatin and 5‐fluorouracil. Tumour formation was monitored by calliper measurements. Physical activity was continuously monitored by infrared imaging. *Ex vivo* muscle performance was determined by myography, muscle fatty acid composition by gas chromatography, and plasma cytokine levels by Luminex xMAP technology. Patient‐derived CRC organoids and C2C12 myotubes were used to determine whether SNC affects chemotherapy sensitivity *in vitro*.

**Results:**

Specific nutritional composition increased muscle contraction capacity of chemotherapy‐treated tumour‐bearing mice (*P* < 0.05) and enriched ω‐3 fatty acid composition in muscle without affecting treatment efficacy (*P* < 0.0001). Mice receiving SNC maintained physical activity after chemotherapy and showed decreased systemic inflammation. Therapeutic response of CRC organoids was unaffected by SNC nutrients, while cell viability and protein synthesis of muscle cells significantly improved.

**Conclusions:**

The results show that specialized nutritional support can be used to maintain muscle function and physical activity levels during chemotherapy without increasing tumour viability. Therefore, nutritional strategies have potential value in promoting cancer and chemotherapy tolerance.

## Introduction

Colorectal cancer (CRC) is the third most commonly diagnosed cancer and accounts for 9.2% of total cancer deaths, which makes CRC the second leading cause of cancer‐related death worldwide.^1^ Approximately half of the patients with advanced stage CRC suffer from cancer cachexia.^2^, ^3^ Cancer cachexia contributes to ~20% of all cancer deaths^2^ and is associated with physical disability, systemic inflammation, metabolic abnormalities, poor quality of life, and poor survival.^4^ Cachexia is defined as ‘a multifactorial syndrome defined by an ongoing loss of skeletal muscle mass that cannot be fully reversed by conventional nutritional support and leads to progressive functional impairment’.^3^ It is a metabolic disorder in which a negative energy balance is caused both by tumour‐induced and chemotherapy‐induced reprogramming of energy metabolism and by a reduced food intake.^5^ Tumour‐derived factors promote the systemic release of metabolites from amino acid reservoirs, such as skeletal muscle and adipose tissue, which tumour cells subsequently exploit for their growth.^6^

Cancer patients receiving chemotherapy for the treatment of (metastatic) CRC may experience a further deterioration of their performance status compared with pretreatment, which interferes with treatment completion and efficacy.^7^ Key elements in this process include reduced physical activity and a reduction of muscle mass and performance caused by chemotherapy‐induced muscle damage and fatigue.

Therapeutic approaches that are aimed at prolonging ‘disease‐tolerant’ states are actively studied in infectious diseases but rarely in cancer. ‘Cancer tolerance’ would be associated with maintenance of muscle function, an improved quality of life, and an increased capacity to endure chemotherapy. Providing anabolic support to muscle tissue seems a promising strategy to boost the ability of the patients to engage in physical activity and thereby improve tolerance to cancer and chemotherapy. Nutritional support is part of daily clinical practice for patients with increased nutritional need. Specialized nutritional support with a specific nutritional composition (SNC) has proven to initiate anabolic stimulation in sarcopenic and cachectic patient populations.^8^, ^9^, ^10^, ^11^, ^12^ As it is hypothesized that combining multiple specific nutrients leads greater impact on muscle atrophy, a novel SNC was designed.^13^ This novel *n*‐3 polyunsaturated fatty acid (PUFA) [eicosapentaenoic acid (EPA) and docosahexaenoic acid (DHA)] and leucine‐enriched, high‐protein (100% whey protein), energy‐dense SNC that contains additional vitamin D and a specific mixture of prebiotic fibres is expected to provide anabolic stimulation of muscle in cancer patients. We hypothesize this SNC will provide benefit of muscle outcomes while the effects of anti‐cancer treatment remain unaffected.^14^, ^15^, ^16^

In the present study, we used *in vivo* and *in vitro* models for aggressive CRC to test the effects of specialized nutritional support on muscle performance and physical activity during chemotherapy treatment, while also assessing the effects of such support on anti‐tumour treatment efficacy.

## Methods

### Ethical approval

This study was conducted in accordance with institutional guidelines for the care and use of laboratory animals, and all animal procedures related to the purpose of the research were approved by the Animal Welfare Body under the ethical licence of University Utrecht, Medical Center Utrecht, the Netherlands, as filed by the national competent authority, securing full compliance the European Directive 2010/63/EU for the use of animals for scientific purposes, and have therefore been performed in accordance with the ethical standards laid down in the 1964 Declaration of Helsinki and its later amendments.

### *In vivo* C26 tumour model

Eighty‐two 5‐ to 6‐week‐old male CD2F1 mice with body weight around 20 g at arrival were purchased at Charles River Laboratories (Calco, Italy) and housed individually (due to physical activity tracking) in Type 3 cages under standard conditions (20–24°C and humidity between 45% and 60%) at GDL Utrecht (Utrecht, the Netherlands), with 12 light (7 a.m.)/12 dark cycle (7 p.m.). Upon arrival, mice were randomized in five groups based on body weight: non‐tumour bearing (non‐TB) (*n* = 10), TB (*n* = 18), TB with chemotherapy (*n* = 18), TB with SNC (*n* = 18), and TB with SNC and chemotherapy (*n* = 18). Mice were allowed to acclimatize for 1 week and were fed *ad libitum* regular control AIN93‐M diet (ssniff‐Spezialdiäten GmbH, Soest, Germany), which was substituted with specialized nutritional support at the day of tumour inoculation. Researchers were blinded to diet and treatment identity. Prior to tumour inoculation, C26 cells were trypsinized, resuspended in sterile 1× phosphate‐buffered saline (PBS; Thermo Fisher Scientific, Breda, The Netherlands), and injected subcutaneously in the right flank using 1.0 × 10^6^ cells in 100 μL of Hank's balanced salt solution (Life Technologies, Bleiswijk, The Netherlands). Non‐TB mice were sham injected with 100 μL Hank's balanced salt solution. Tumour development was monitored by measuring volume with a calliper. Tumour volume was calculated by formula 0.5 (length × width^2^). At Day 8, a single dose of 100 μL of 25 mg/kg oxaliplatin and three doses of 25 mg/kg 5‐fluorouracil (Days 8, 10, and 12) or vehicle (NaCl solution + 0.9% glucose) were administered via intraperitoneal injection. A novel SNC designed to support muscle mass and function was translated into animal feed by producing an AIN93‐M‐based semi‐synthetic modified diet (ssniff‐Spezialdiäten GmbH) with an isocaloric, although not isonitrogenous control diet. The composition of the SNC and control diet is shown in *Table* 1. As quality control, diets were checked by analysing protein and fatty acid content (data not shown).

**Table 1:** Dietary composition of control and SNC diet

	Control diet (g/kg)	SNC (g/kg)
**Carbohydrates**		
Cornstarch	604.2	492.1
Sucrose	97.30	92.80
Fibre (cellulose)	47.50	22.00
GOS	0.00	40.00
FOS	0.00	2.20
**Protein**		
Casein	140.00	0.00
Whey	0.00	237.6
l‐Leucine	0.00	8.60
Isoleucine (2:1:1)	0.00	2.50
Valine (2:1:1)	0.00	3.50
**Fat**		
Fish oils	0.00	24.80
EPA	0.00	6.9
DHA	0.00	3.0
Soybean oil	38.63	15.20
**Other**		
Mineral mix	35.00	35.00
Vitamin mix	10.00	10.00
Vitamin D	1000.00	5000.00
Choline bitartrate	2.50	2.50
tBHQ	0.008	0.008
**Total nutritional value**		
Total carbohydrates	688.50	591.70
Total protein	123.40	215.70
Total fat	40.90	40.90
**Other specifications**		
Kcal	3616.00	3600.00
Total weight (g)	1000.00	999.91
Protein energy percentage (EN%)	13.66	23.80

DHA, docosahexaenoic acid; EPA, eicosapentaenoic acid; FOS, fructooligosaccharides; GOS, galactooligosaccharides; SNC, specific nutritional composition; tBHQ, *tert*‐butylhydroquinone.

Physical activity was registered by in‐cage infrared camera imaging (Dual Technology Detector DUO 240, Visonic (Tel Aviv, Israel); adapted by R Visser, Netherlands Institute for Neurosciences) and recorded with MED‐PC IV software (Med Associates, Hertfordshire, United Kingdom). Activity was expressed in mean counts per day for the total 24 h period and the light period (inactive period) separately, per individual mouse, and was expressed relative to baseline activity (Day 2). Actograms present raw activity of the biological rhythm averaged per group.

Mice were sacrificed at Day 20 after inoculation by cardiac puncture followed by cervical dislocation under total anaesthesia (isoflurane/N_2_O/O_2_), and the skeletal muscles from the hind limb (tibialis anterior, extensor digitorum longus, soleus, plantaris, and gastrocnemius muscles), spleen, and tumour were dissected, weighed, and stored at −80°C until further use.

### *In vitro* treatments

Conventional CRC chemotherapeutics were tested *in vitro*: oxaliplatin (S1224, Selleckchem, Huissen, The Netherlands), 5‐fluorouracil (F6627, Sigma‐Aldrich, Zwijndrecht, The Netherlands), irinotecan (S1198, Selleckchem), or a combination of oxaliplatin and 5‐fluorouracil (OXF). All chemotherapeutics were dissolved in dimethyl sulfoxide (DMSO).

### *Ex vivo* muscle performance

At Day 20, *ex vivo* muscle performance was assessed. In short, directly after isolation, the left musculus extensor digitorum longus was positioned in an organ tissue bath (Hugo Sachs Elektronik, March, Germany), which was filled with Krebs–Heinselet buffer (mM: NaCl 118, KCl 4.75, MgSO_4_ 1.18, CaCl_2_ 2.5, KH_2_PO_4_ 1.17, NaHCO_3_ 24.9, and glucose 10). The buffer was kept at 30°C and continuously gassed with 95% O_2_ and 5% CO_2_. The distal end of the muscle was connected to the base of the tissue bath, whereas the proximal end was attached to a force transducer (F30, Hugo Sachs Elektronik). The muscle was positioned between two platinum electrodes for electrical stimulation. Isometric force signals of the force–frequency (Hz) curve were analysed for maximal and total force, contraction, and relaxation velocity and times. The maximal force production was followed in time during an exercise protocol, where 100 stimuli at the maximal force production gave insight in contraction force after simulation of fatigue due to long‐term contraction.

### Fatty acid analysis

For fatty acid analyses, in short, the musculus plantaris was weighted and cryo‐desiccated. Muscle samples were resuspended in weight‐adjusted volume of 2% perchloric acid solution and homogenized. Blood samples were centrifuged. All samples were analysed with gas chromatography as described previously.^17^ Values are represented as relative fatty acid content (% of total fatty acid) and as ratio of omega‐6 divided by omega‐3 PUFA.

### Luminex assay

Cytokine levels were determined in blood plasma (collected via cardiac puncture in heparin‐coated tubes) using a multiplex immunoassay (MCYTOMAG‐70K, Merck Millipore, Amsterdam, The Netherlands) based on Luminex xMAP technology at the MultiPlex Core Facility of the Laboratory of Translational Immunology, University Medical Centre Utrecht (Utrecht, the Netherlands). Values are represented as relative plasma concentrations compared with TB mice fed with the control diet.

### Tumour organoid and cell culture

Patient‐derived CRC organoids were previously established and characterized.^18^ CRC organoids were cultured in droplets of Reduced Growth Factor Basement Membrane Extract (BME; Amsbio, Abingdon, United Kingdom), and medium (Supporting Information, *Data* S1) was refreshed twice a week or at indication. Organoids were passaged through TrypLE Express (Invitrogen, Breda, The Netherlands) treatment combined with mechanic disruption.

Murine colon adenocarcinoma cell line C26 was cultured in adherent plates and maintained in Dulbecco's modified Eagle's medium (DMEM) high glucose (Invitrogen), supplemented with 1% penicillin–streptomycin, 1% GlutaMAX, and 10% heat‐inactivated foetal bovine serum (Invitrogen). C2C12 mouse myoblasts (kindly provided by Maastricht University^19^) were maintained in growth medium containing DMEM high glucose (Sigma‐Aldrich), supplemented with 1% penicillin–streptomycin, 2.4 g/L NaHCO_3_, and 10% foetal bovine serum (growth medium, Invitrogen). Myoblasts were cultured twice a week until maximal confluency of 70%. To generate myotubes, myoblasts were grown in differentiation medium containing DMEM with 2.4 g/L NaHCO_3_, 1% penicillin–streptomycin, and 2% horse serum (Gibco, Breda, The Netherlands).

All cultures were maintained at 37°C in a humidified atmosphere containing 5% CO_2_ and have repeatedly been tested negative for *Mycoplasma*.

### Specialized nutritional support *in vitro*

The SNC consists of four key nutritional ingredients (SNCi) that allowed *in vitro* testing: a final dosing of 1 mM l‐leucine (#61819, Sigma‐Aldrich), 10 nM vitamin D_3_ (D1530, Sigma‐Aldrich), 25 μM EPA (E7006, Sigma‐Aldrich), and 12.5 μM DHA (D2534, Sigma‐Aldrich). Leucine was dissolved in PBS–Tween (0.1%); EPA, DHA, and vitamin D_3_ were dissolved in DMSO for organoid screenings. For muscle cells assays, EPA and DHA were dissolved in 96% ethanol and further diluted in PBS + 2.5% bovine serum albumin (fatty acid free), and vitamin D_3_ was dissolved in 96% ethanol and leucine in differentiation medium.

### Cell viability of C2C12 myotubes

C2C12 myoblasts were differentiated in 5 days into myotubes in 96‐well plates at 1.0e5 cells/mL (100 μL per well) and were pre‐exposed for 1 h to SNCi, before exposure to OXF (1:1 ratio) for 24 h. Concentration series were tested six‐fold (*n* = 3). Cell viability was assessed using CellTiter‐Glo^®^ 2.0 luminescence assay (Promega, Leiden, The Netherlands). Values are expressed as ratio to control cells receiving only vehicle.

### Basal protein synthesis of C2C12 myotubes

Basal myotube protein synthesis was determined using an adapted version of the ‘SUnSET’ protocol (*Data S1*).^20^, ^21^ In short, after 48 h incubation with SNCi and 24 h treatment with OXF, myotubes were deprived of l‐leucine and serum for 4 h and incubated overnight with anti‐puromycin antibody (clone 12D10, 1:200, Merck Millipore) and secondary antibody (Anti‐mouse DyLight 488, 1:200, Molecular Probes, Breda, The Netherlands) for 1 h. Fluorescence was measured, and protein content was determined using the amido black assay.^22^ Values are expressed as the ratio to control cells receiving only vehicle.

### High‐throughput screening of patient‐derived colorectal cancer organoids

Colorectal cancer organoids were cultured as described earlier. A bottom layer of 10 μL BME was dispensed in a 384‐well plate (Costar^®^ CLS3571; Corning, Amsterdam, The Netherlands) using the Multidrop™ Combi Reagent Dispenser with a small tubing cassette (Thermo Fisher Scientific), centrifuged, and kept at room temperature for 15–25 min to allow full polymerization of BME. Depending on proliferation rate, approximately 750–1000 three‐day‐old CRC organoids suspended in 40 μL basal culture medium were dispensed on top of BME and briefly centrifuged (100 g at 4°C). Organoids settled for 4 h before exposure to nutritional factors and 24 h later exposed to chemotherapeutics. Both nutritional factors and chemotherapeutics were added using HP D300e digital dispenser (Tecan, Männedorf, Switzerland), and non‐treated wells received normalization with DMSO. After exposure to SNCi (96 h) and chemotherapy (72 h), tumour organoid viability was quantified using CellTiter‐Glo 2.0 luminescence assay (Promega) and SpectraMax M5e reader (Molecular Devices, San Jose, California, United States of America). For each organoid, experiments were executed at least three times, with a minimum of three replicates per experiment. Data were analysed using R software using the packages nlme (Version 3.1‐124) and lme4 (Version 1.1‐11) for R (Version 3.2.1 for macOS).

To estimate the average 50% inhibitory concentration (IC_50_) for each screened drug (5‐fluorouracil, oxaliplatin, and irinotecan) in human organoids in general with or without addition of SNCi, all data points were combined from repeated experiments for all five human organoid lines for each drug, normalized per plate to the zero‐concentration treatment control condition (set at 100% viability). Three separate four‐parameter non‐linear mixed‐effect models—for each drug one—were used to fit dose–response curves (*Data* S1).

### Statistical analyses

Sample size of experimental groups (*n* = 18) was based on the *ex vivo* muscle function parameter using power (*β*) = 0.8, two‐sided effect (*α*) = 0.05, four groups and comparisons, and two factors (treatment and diet), including risk of drop out caused by tumour development. Sample size of control group (*n* = 10) was based on previously obtained data. Sample sizes can differentiate between assays due to limited equipment or sample or technical limitations. All results are displayed as mean ± standard error of the mean. *Ex vivo* skeletal muscle performance data and repeated measurements were analysed by mixed models with *post hoc* Fisher's least significant difference. All single time‐point data were statistically analysed by using two‐way analysis of variance with *post hoc* least significant difference. For statistical analysis of high‐throughput screens, see section earlier. Statistical analyses were performed using SPSS 19.0, and figures were created using GraphPad Prism 8 Version 8.4.0 and Adobe Illustrator Version 24.3. All statistical differences were considered significant at a *P*‐value <0.05.

## Results

The C26 colorectal cancer cell line is widely used in muscle wasting studies^23^ and was also used in the current *in vivo* study to investigate the effect of specialized nutritional support on muscle function, tumour growth, inflammation, physical activity, and response to chemotherapy treatment. Tumour growth was initiated by subcutaneous injection of C26 cells at Day 0. Chemotherapy treatment consisted of a single dose of oxaliplatin (Day 8), followed by three doses of 5‐fluorouracil (Days 8, 10, and 12). Food intake was monitored and did not differ between groups over (*Figure* S1A and S1B). Upon sacrifice at Day 20, the effects of the SNC and chemotherapy treatment on *ex vivo* muscle function were assessed.

### Specialized nutritional support preserves muscle performance during chemotherapy treatment in C26 tumour‐bearing mice

**Figure 1:**
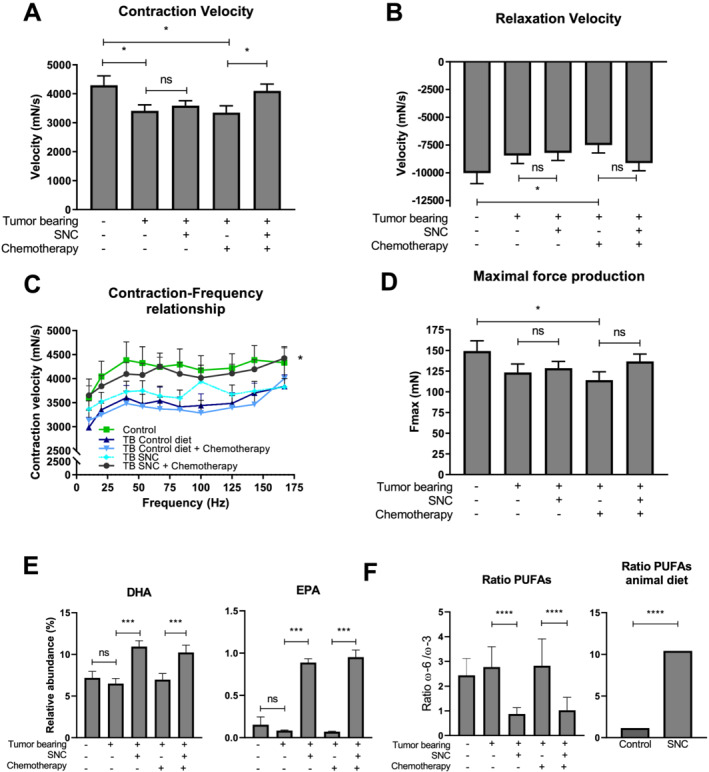
Specialized nutritional support preserves muscle performance during chemotherapy. *Ex vivo* muscle function of musculus extensor digitorum longus and fatty acid composition of musculus plantaris. Non‐tumour‐bearing (TB) control *n* = 9, TB control diet *n* = 18, TB control diet + chemotherapy *n* = 18, TB specific nutritional composition (SNC) *n* = 17, and TB SNC + chemotherapy *n* = 17. (*A*) Contraction velocity (mN/s) of muscle contraction at 83 Hz. (*B*) Relaxation velocity of muscle contraction (mN/s) at 83 Hz. (*C*) Contraction–frequency relationship for contraction velocity (mN/s). (*D*) Maximal force production during tetanic force measurement at 83 Hz. (*E*) Relative abundance (%) of docosahexaenoic acid (DHA) (left) and eicosapentaenoic acid (EPA) (right) in musculus plantaris. (*F*) Omega‐6/omega‐3 ratios reflecting the fatty acid composition of musculus plantaris (left panel) and SNC and control diet animal feed (right panel). Force–frequency relationships were statistically tested using mixed model analysis, *post hoc* least significant difference. All other comparisons were statistically tested using one‐way analysis of variance, *post hoc* least significant difference. Significant differences are shown as *P < 0.05, **P < 0.01, ***P < 0.001, and ****P < 0.0001. PUFAs, polyunsaturated fatty acids.

Contraction and relaxation velocity are measurements of muscle performance and health; they represent the contractile properties of the muscles in response to nerve innovation. TB mice showed a significant reduction of muscle contraction (*Figure* 1A) and relaxation velocity (*Figure* 1B) compared with non‐TB mice, indicating impaired muscle function. Chemotherapy treatment did not significantly further deteriorate muscle performance. SNC significantly improved contraction velocity in chemotherapy‐exposed TB mice when compared with TB mice fed with the control diet. The contraction and relaxation velocity were restored to levels of non‐TB mice, which did not receive chemotherapy treatment (*Figure* 1A and 1B). The SNC also significantly rescued the maximum contraction velocity at different stimulation frequencies (Hz) to levels observed in non‐TB mice that did not receive chemotherapy treatment (*Figure* 1C). Moreover, the maximal force produced by the muscle followed the same trends as contraction and relaxation velocity, where the SNC restored maximal force in chemotherapy‐exposed TB mice when compared with mice fed with the control diet (*Figure* 1D). Body weight development over time and muscle mass at time of sacrifice are shown in *Figure* S2A and S2B. The fatty acid composition of muscle, focusing on PUFAs, beneficial ω‐3 fatty acids, such as DHA and EPA, and disadvantageous chronic inflammation‐induced ω‐6 fatty acids, such as arachidonic acid and linoleic acid, was used to assess local effects of the SNC.^24^ The SNC caused a significant increase in both DHA and EPA abundance compared with control‐fed TB mice (*Figure* 1E). A diet high in omega‐6/omega‐3 ratio represents a pro‐inflammatory PUFA status, whereas a low ratio is associated with an anti‐inflammatory, healthy PUFA status. The SNC significantly decreases omega‐6/omega‐3 ratios compared with control‐fed TB mice in both untreated and chemotherapy‐treated TB mice in muscle (*Figure* 1F) and blood (data not shown). Moreover, comparing the animal diets, animals fed with the SNC show significantly decreased omega‐6/omega‐3 ratio in muscle (*Figure* 1F). The aforementioned results indicate that specialized nutritional support containing PUFAs directly changes fatty acid profile to a beneficial ratio for muscular function.

### Specialized nutritional support does not interfere with chemotherapy treatment efficacy in C26 tumour‐bearing mice

**Figure 2:**
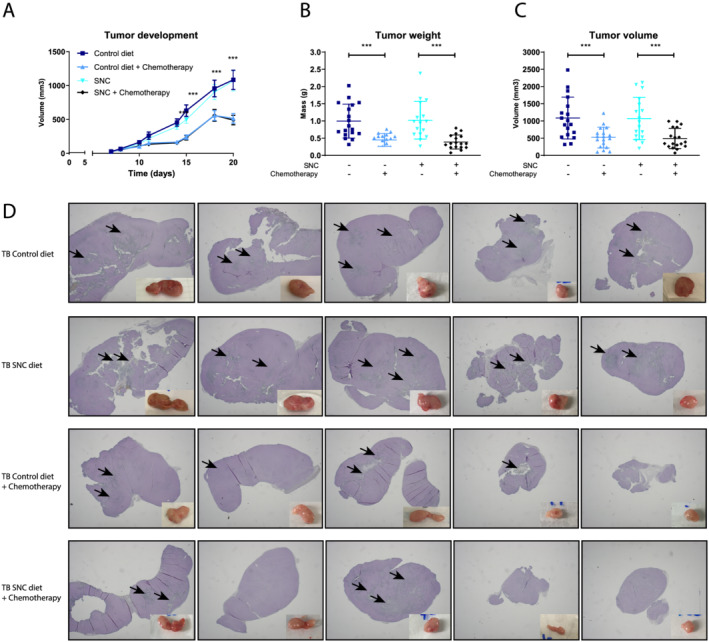
Specialized nutritional support does not interfere with chemotherapy efficacy. (*A*) Subcutaneous tumour development (mm^3^) over time, measured by calliper. (*B*) Tumour weight (g) measured at sacrifice (Day 20). (*C*) Tumour volume (mm^3^) measured at sacrifice (Day 20). (*D*) Images of fresh tumour tissues per group and histological slides (haematoxylin and eosin staining), black arrows indicating necrotic areas. Tumour‐bearing (TB) control diet *n* = 18, TB control diet + chemotherapy *n* = 18, TB specific nutritional composition (SNC) *n* = 18, and TB SNC + chemotherapy *n* = 18. Repeated measurements were statistically tested using mixed model analysis, *post hoc* least significant difference. Single time‐point comparisons were statistically tested using one‐way analysis of variance, *post hoc* least significant difference. Significances are shown as *P < 0.05, **P < 0.01, and ***P < 0.001.

To investigate potential interactions of the SNC with the efficacy of chemotherapy treatment, tumour development was monitored by volumetric measurements throughout the study. Chemotherapy treatment (administered from Days 8 to 12) resulted in a significant decrease in tumour volume over time from Day 14 onwards comparing chemotherapy‐treated to untreated TB groups, regardless of nutritional support (*Figure* 2A). At time of sacrifice (Day 20), both tumour volume (*Figure* 2B) and tumour mass (*Figure* 2C) significantly differed between chemotherapy‐treated and untreated TB mice, independent of the diet fed. These data show that nutritional support does not interfere with chemotherapy treatment efficacy. In *Figure* 2D, five pictures of representative tumours are shown from each experimental group, indicating clear differences in tumour development between chemotherapy‐treated and untreated groups, whereas the SNC had no significant effect on tumour appearance and size. As indicated by black arrows, necrosis was found in almost all specimens, regardless of therapy or diet, and therefore most likely only related to size of tumours.

### Specialized nutritional support normalizes systemic inflammation during chemotherapy in C26 tumour‐bearing mice

**Figure 3:**
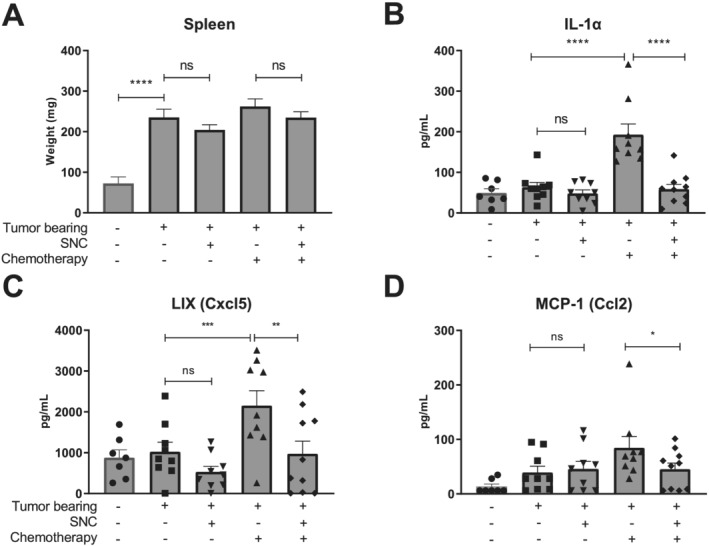
Specialized nutritional support normalizes systemic inflammation during chemotherapy. (*A*) Splenic weight (mg) at sections (Day 20), representative for degree of splenomegaly. Non‐tumour‐bearing (TB) control *n* = 10, control diet *n* = 18, control diet + chemotherapy *n* = 18, specific nutritional composition (SNC) *n* = 17, and SNC + chemotherapy *n* = 18. (*B*) Blood plasma expression levels of interleukin‐1α (IL‐1α) in g/mL. (*C*) Blood plasma expression levels of LIX (Ccl5) in pg/mL. (*D*) Blood plasma expression levels of monocyte chemoattractant protein‐1 (MCP‐1) (Ccl2) in pg/mL. Non‐TB control *n* = 7, TB control diet *n* = 9, TB control diet + chemotherapy *n* = 9, TB SNC *n* = 9, and TB SNC + chemotherapy *n* = 10. Comparisons were statistically tested using one‐way analysis of variance, *post hoc* least significant difference. Significant differences are shown as *P < 0.05, **P < 0.01, ***P < 0.001, and ****P < 0.0001.

Cancer cachexia is known to be associated with systemic inflammation.^25^ Dietary enrichment of EPA and DHA omega‐3 fatty acids is known to reduce systemic inflammation by decreasing the production of the inflammatory mediator PGE2.^26^ Nutritional support aims to mitigate systemic inflammatory responses and cytokine production caused by presence of cancer cachexia, tumour formation, and chemotherapy treatment. One of the hallmarks of tumour‐induced inflammation is splenomegaly,^27^ which we observed in all TB mice regardless of chemotherapy treatment (*Figure* 3A), while non‐TB mice maintained a healthy spleen weight. Comparing chemotherapy‐exposed TB mice fed with SNC to control‐fed mice, a trend towards significance was observed towards decreased splenomegaly with nutritional support.

To assess systemic inflammation, blood plasma samples were analysed for interleukin‐1α (IL‐1α), LIX (mouse Cxcl5), and monocyte chemoattractant protein‐1 (MCP‐1) levels using Luminex xMAP technology. Several cytokines have been implicated in systemic inflammation during cancer cachexia and CRC. This includes IL‐1α, a master regulator of inflammation, which is linked to an increased risk of muscle wasting in cancer cachexia.^28^ LIX (Cxcl5), which acts as a neutrophil chemoattractant, has been correlated with worse prognosis of CRC patients.^29^ The cytokine MCP‐1 (Ccl2) signals macrophage and monocyte migration and infiltration and was recently identified as a prognostic marker in pre‐cachectic pancreatic cancer patients.^30^ Chemotherapy‐treated TB mice fed with control diet showed a significant increase in circulating IL‐1α, LIX, and MCP‐1 levels when compared with untreated TB mice fed with control diet (*Figure* 3B–3D). Specialized nutritional support normalized the expression of IL‐1α, LIX, and MCP‐1 to levels observed in non‐treated non‐TB mice (*Figure* 3B–3D). Here, the SNC normalizes expression levels of chemokine LIX compared with control‐fed TB mice. In *Figure* 3D, upon chemotherapy treatment, MCP‐1 levels significantly rise in control‐fed TB mice, whereas the SNC significantly neutralizes the expression compared with control diet.

### Specialized nutritional support restores physical activity after chemotherapy treatment cycles in C26 tumour‐bearing mice

**Figure 4:**
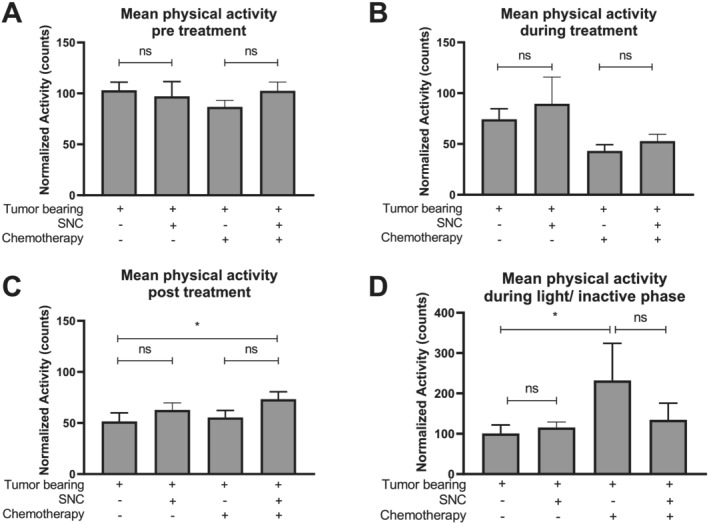
Specialized nutritional support restores physical activity after chemotherapy treatment cycles. (*A*) Mean physical activity before chemotherapy treatment (Day 4) relative to Day 2 (set to 100%) physical activity as baseline. (*B*) Mean physical activity during chemotherapeutic treatment (Day 12) relative to Day 2 (set to 100%) physical activity as baseline. (*C*) Mean physical activity after chemotherapeutic treatment (Day 19) relative to Day 2 (set to 100%) physical activity as baseline. (*D*) Mean physical activity in the inactive light period relative to Day 2 (set to 100%). Tumour‐bearing (TB) control diet *n* = 14, TB control diet + chemotherapy *n* = 16, TB specific nutritional composition (SNC) *n* = 12, and TB SNC + chemotherapy *n* = 15. Statistical differences were tested using one‐way analysis of variance *post hoc* least significant difference. Significances are shown as *P < 0.05.

Before onset of tumour formation and start of treatment, baseline physical activity showed no differences between groups (*Figure* 4A). Chemotherapy treatment caused a non‐significant decrease in physical activity comparing treated TB mice with non‐treated TB mice, independent of diet (control or SNC) (*Figure* 4B). During chemotherapy treatment, the SNC did not significantly affect physical activity compared with the control diet. We observed that after treatment, chemotherapy‐treated TB mice fed with SNC showed increased physical activity compared with chemotherapy‐treated control‐fed TB mice (*Figure* 4C). Moreover, non‐treated TB mice fed with SNC showed a non‐significant increase in physical activity compared with control‐fed TB mice. Overall, specialized nutritional support after chemotherapy treatment restored physical activity towards baseline physical activity levels of non‐TB animals. Physical activity levels in the light (inactive) phase are known to be a measure of discomfort and distress.^31^ Control‐fed TB mice treated with chemotherapy showed increased physical activity during the light phase compared with control‐fed TB mice without chemotherapy treatment (*Figure* 1D). Chemotherapy‐treated TB mice fed with SNC showed decreased light phase physical activity compared with control‐fed TB mice, indicating restored well‐being. The total physical activity is reflected in an actogram, which shows physical activity per group over the time course of the whole experiment, including non‐TB control mice (*Figure* S3).

### Specific nutritional composition ingredient maintains anabolic capacity in muscle cells exposed to chemotherapy *in vitro*

**Figure 5:**
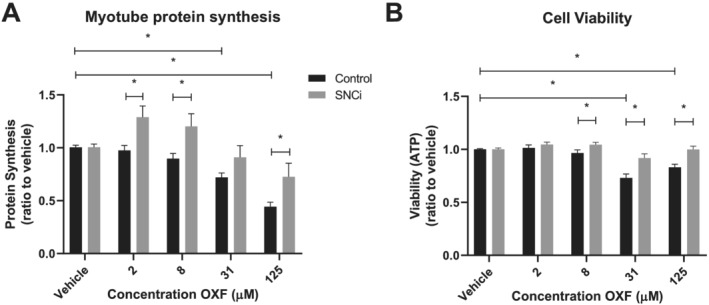
Specific nutritional composition ingredient (SNCi) maintains anabolic capacity in muscle cells exposed to chemotherapy *in vitro*. (*A*) Myotube protein synthesis ratio of chemotherapy‐treated C2C12 myotubes after exposure to SNCi (grey bars) or control (black bars). Myotubes were differentiated for 5 days before chemotherapy exposure of 24 h, after 24 h before exposure to nutritional support. (*B*) Cell viability of chemotherapy‐treated C2C12 myotubes after exposure to nutritional support (grey bars) or control (black bars). Chemotherapy exposure of 24 h, after 1 h before exposure to nutritional support. All values are expressed as ratio to vehicle (vehicle set to 100%). Comparisons were statistically tested using one‐way analysis of variance, *post hoc* least significant difference. Significances are shown as *P < 0.05, **P < 0.01, and ***P < 0.001.

To investigate direct effects of SNCi and chemotherapy treatment on skeletal muscle, differentiated murine C2C12 myotubes were used. First, it was investigated whether chemotherapy exposure would result in loss of basal protein synthesis in myotubes and to which extent this could be rescued by specialized nutritional support. As shown in *Figure* 5A, SNCi stimulated myotube protein synthesis in the presence of all chemotherapy concentrations tested. Myotube protein synthesis decreased after exposure to chemotherapy, where addition of SNCi rescued this detrimental effect. Furthermore, the effects of chemotherapy and SNCi on ATP levels, as a measure for myotube cell viability (*Figure* 5B), were investigated. Overall, cell viability of myotubes was negatively impacted by chemotherapy. SNCi with chemotherapy showed a significant rescuing effect compared with vehicle in 8 and 31 μM. At higher chemotherapy concentrations (125 μM), SNCi was no longer able to counteract the treatment effects on muscle cell viability.

### Specific nutritional composition ingredient does not interfere with tumour organoid killing by chemotherapy *in vitro*

**Figure 6:**
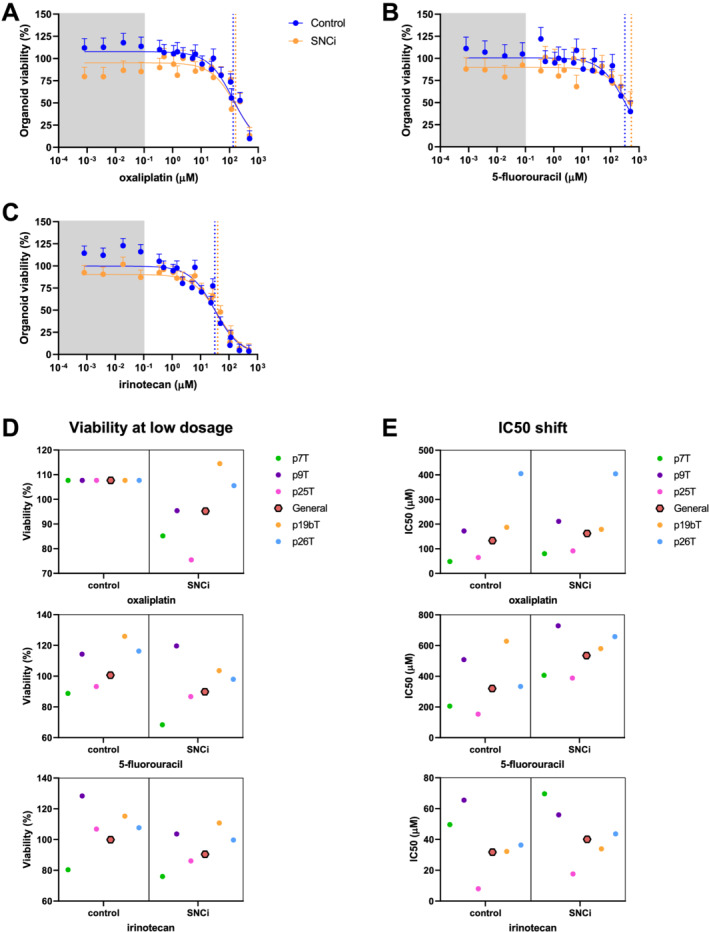
Specific nutritional composition ingredient (SNCi) does not interfere with tumour organoid killing by chemotherapy *in vitro*. Average effects on five patient‐derived organoids, all toxicity assays were executed at least as *n* = 3. Blue lines represent control, and orange lines represent SNCi. Grey area represents low dosage range. (*A*) Dose–response curves of general effect of oxaliplatin treatment in absence or presence of SNCi. (*B*) Dose–response curves of general effect of 5‐fluorouracil treatment in absence or presence of SNCi. (*C*) Dose–response curves of general effect of irinotecan treatment in absence or presence of SNCi. (*D*) Variability of cell viability at low dosage of chemotherapy (top to bottom; oxaliplatin, 5‐fluouracil, and irinotecan) for individual colorectal cancer (CRC) organoids with SNCi (right panel) compared with control (left panel), represented relative to control viability (non‐treated). NB: oxaliplatin without nutritional support; present variation is not detected by non‐linear mixed model; random intercepts per organoid were estimated as null. (*E*) Variability of shift in IC_50_ values for individual CRC organoids with SNCi (right panel) compared with control (left panel).

The earlier results show that SNC does not influence chemotherapy efficacy in C26 TB mice (*Figure* 2). Human patient‐derived CRC organoids can be used as an *in vitro* model system to assess individual patient response to chemotherapy.^32^ To evaluate whether SNCi interferes with the efficacy of chemotherapy in CRC, a panel of five patient‐derived CRC organoids was used, reflecting the heterogeneous CRC patient population. All CRC organoids were exposed to concentration series of the three most commonly used chemotherapeutics in the systemic treatment of CRC (oxaliplatin, 5‐fluorouracil, and irinotecan) in the presence or absence of SNCi. Prior to 72 h chemotherapy exposure, CRC organoids were pre‐exposed for 24 h with SNCi before cell viability was measured. All toxicity assays for all organoids were performed at least three times. To assess the overall effect of SNCi on chemotherapy efficacy in CRC, the data obtained from all toxicity assays on all CRC organoids were combined into one large composite dataset containing more than 3400 data points. On this aggregate level, SNCi had no significant effect on the mean IC_50_ values (*Table* S1) of CRC organoids for oxaliplatin (*Figure* 6A), 5‐fluorouracil (*Figure* 6B), or irinotecan (*Figure* 6C). These findings are in line with *in vivo* tumour development data of C26 TB mice presented in *Figure* 2A–2C.

Variability of IC_50_ values for the three drugs between individual patient‐derived CRC organoid responses was observed, possibly reflecting the heterogeneity of tumour responses that are observed in clinical practice. Also when analysed at the level of individual organoids, IC_50_ values for all three drugs were not significantly affected by SNCi (data not shown). Addition of SNCi appeared to decrease organoid viability in the lower range of chemotherapy concentrations for all three drugs (*Figure* 6A–6C), although these effects were not significant when analysing the aggregate data or when analysing the data of individual organoids (*Figure* 6D). These data show that specialized nutritional support does not interfere with chemotherapy efficacy in a series of human CRC organoids, which confirms our findings in C26 TB mice.

## Discussion

In this study, we show that a novel ω‐3 PUFA (EPA and DHA) and leucine‐enriched, high‐protein (100% whey protein), energy‐dense SNC, which contains additional vitamin D and a specific mixture of prebiotic fibres, improves muscle performance and maintains physical activity of chemotherapy‐treated mice while decreasing systemic inflammation *in vivo*. Moreover, specialized nutritional support increased *in vitro* muscle protein synthesis and cell viability during chemotherapy treatment. Indeed, current evidence from clinical trials suggests an overall positive effect of high‐protein and leucine, PUFA‐enriched nutritional interventions, resulting in lean body mass gain and improved overall quality of life in several cancer types.^33^, ^34^, ^35^ However, the direct effects of combined nutritional support strategies on tumour development and their role in treatment toxicity and resistance remained unanswered.^36^, ^37^ In this study, specialized nutritional support does not interfere with chemotherapy treatment efficacy of patient‐derived CRC organoids. Furthermore, the direct effects of this SNC during chemotherapy treatment were tested *in vivo*, providing evidence that in addition to the beneficial effects on muscle function and quality of life, nutritional support does not enhanced tumour proliferation or altered therapy response.

Reduced skeletal muscle mass is recognized as a major predictor of poorer prognoses and outcomes in cachectic cancer patients.^3^ Additionally, altered muscle composition can be observed in cachectic cancer patients, which can result in deterioration of muscle performance. However, muscle performance itself is often overlooked and in our opinion is an equally important component of muscle function, thus quality of life, compared with solely quantifying skeletal muscle mass. In this study, we show that specialized nutritional support is able to restore muscle performance after chemotherapy treatment compared with mice fed with the control diet. By supplementing anabolic factors, this novel SNC has the capacity to limit functional damage to muscle after exposure to chemotherapy treatment.

Various systematic reviews have evaluated the efficacy of omega‐3 PUFAs during chemotherapy on patient outcomes in clinical trials, predicting a positive effect on body weight, skeletal muscle mass, quality of life, and performance status.^36^, ^38^ Indeed, our results show that the SNC restores physical activity of TB mice after chemotherapy treatment towards baseline conditions, whereas TB mice fed with the control diet deteriorated in levels of physical activity. Cachectic patients treated with chemotherapy are often severely fatigued, partially due to impaired muscle function, which contributes to decreased physical activity and chemotoxicity. As a result, patients are sometimes forced to discontinue their chemotherapy regimens.^39^ Translating our findings on improved physical activity and circadian rhythm normalization, specialized nutritional support could contribute to improved quality of life of patients and decrease chemotoxicity, enabling treatment continuation and eventually better outcomes. Van Norren *et al*. showed previously that supplementation with single ingredients like extra protein or fatty acids cannot provide benefit.^31^ However, here, we demonstrate that a multifactorial approach in SNC can elicit improved physical activity and muscle performance in chemotherapy‐treated mice by combining leucine‐enriched high‐protein, PUFAs, galactooligosaccharides, fructooligosaccharides, and vitamin D and other specific nutrients.

In Dutch clinical practice, patients who undergo chemotherapy are advised to avoid intake of omega‐3, such as fatty fish or fish oil supplementation in a 48 h window surrounding chemotherapy administration.^40^ A specific subset of omega‐3 fatty acids, platinum‐induced polyunsaturated fatty acids (PIFAs), such as 16:4(*n*‐3) and 12‐oxo‐5,8,10‐heptadecatrienoic acid, are produced by mesenchymal stem cells and are known to enhance platinum‐based chemotherapy resistance in C26 TB mice.^41^ Williard *et al*. found that EPA can be converted to 16:4(*n*‐3) by peroxisomal oxidation.^42^ Because of limited sample of mouse blood plasma, we were unable to determine PIFA levels in this study. Considering no altered platinum‐based chemotherapy response in C26 TB mice was observed in SNC‐fed mice, we hypothesize that in our study, metabolism of ω‐3 fatty acids did not evoke a strong EPA‐mediated production of PIFAs. Additionally, it could be that PIFAs are not as potent at provoking chemoresistance in all cancer types, as emerging clinical evidence in non‐small cell lung cancer patients shows that even with PIFA levels rising, tumour response to platinum‐based chemotherapy treatment and survival is not altered.^43^

Generally speaking, the positive effects of SNC diet were more pronounced in chemotherapy‐treated animals compared with non‐treated animals. In the present study design, we aimed at creating a window of opportunity for nutritional interventions in the pre‐cachectic state in TB mice. Chemotherapy treatment dose was optimized (*unpublished data*) and resulted in slower tumour progression compared with the non‐treated animals, increasing the window of opportunity for nutrition interventions.

To summarize, we have shown that specialized nutritional support increased muscle contraction capacity of chemotherapy‐treated TB mice and improved muscle fatty acid composition without affecting therapy efficacy. TB mice receiving SNC maintained physical activity after chemotherapy. Furthermore, specialized nutritional support decreased systemic inflammation. *In vitro* cell viability of chemotherapy‐treated CRC organoids was unaffected by SNC ingredients. *In vitro* muscle cells showed chemotherapy‐associated toxicity by impaired protein synthesis and cell viability, while SNC ingredients reduced the effects of chemotherapy.

This study shows that an ω‐3 PUFA (EPA and DHA) and leucine‐enriched, high‐protein (100% whey protein), energy‐dense SNC that contains additional vitamin D and a specific mixture of prebiotic fibres can be used to support muscle function and maintain levels of physical activity during chemotherapy without causing tumour protection. Therefore, specialized nutritional support strategies have potential value in promoting tolerance to cancer and chemotherapy in clinical applications. Further clinical evaluation of specialized nutrition is warranted to assess whether these obtained results can be translated to clinical perspectives.

## Funding

This research is part of the SCOPE project (Specialized nutrition to improve outomes of COlorectal cancer PatiEnts) and is supported by the Province of Utrecht, the Netherlands.

## Conflict of interest

F.J.D., H.Q., B.D., M.J.W.F., and M.v.D. are employed at Danone Nutricia Research. L.A.W., D.A.E.R., S.G.E., A.M.M., and O.K. declare that they have no conflicts of interest.

## Supporting information


**Figure S1.** Food intake. (A) Food intake per group at time points *t* = 2, 5, 8, 12, 15, 19. (B) Mean food intake per group

**Figure S2.** Body weight and muscle weights. (A) Body weight (g), expressed as relative body weight to body weight at *t* = 0. (B) Muscle weights (mg) relative to body weight at sacrifice of *m*. *Tibialis* *Anterior*, Extensor *Digitorum Longus*, *m*. *Plantaris*, m. *Gastrocnenius*, m. *Soleus*

**Figure S3.** Actogram of total physical activity over the course of the experiment. (A) Non‐TB mice show very little differences over time, whereas TB mice do show variances in physical activity. (B) During chemotherapy treatment (black box window) physical activity of mice fed with SNC is less impacted compared to control‐fed mice. Moreover, after chemotherapy mice fed with SNC show less disturbances in activity in the light phase compared to control‐fed mice. Non‐TB Control diet *n* = 8, TB Control diet *n* = 14, TB Control diet + chemotherapy *n* = 16, TB SNC *n* = 12, TB SNC + chemotherapy *n* = 15

**Data S1.** Supporting Information

**Table S1.** Delta log IC50 and top viability values of tumor organoid killing curves with corresponding 95% confidence intervals (CI) and p‐values
