# Supplementary figures and images for: Specialized nutrition improves muscle function and physical activity without affecting chemotherapy efficacy in C26 tumour‐bearing mice
Source: J Cachexia Sarcopenia Muscle. 2021 May 6;12(3):796–810. doi: 10.1002/jcsm.12703 (PMC8200448; doi:10.1002/jcsm.12703)

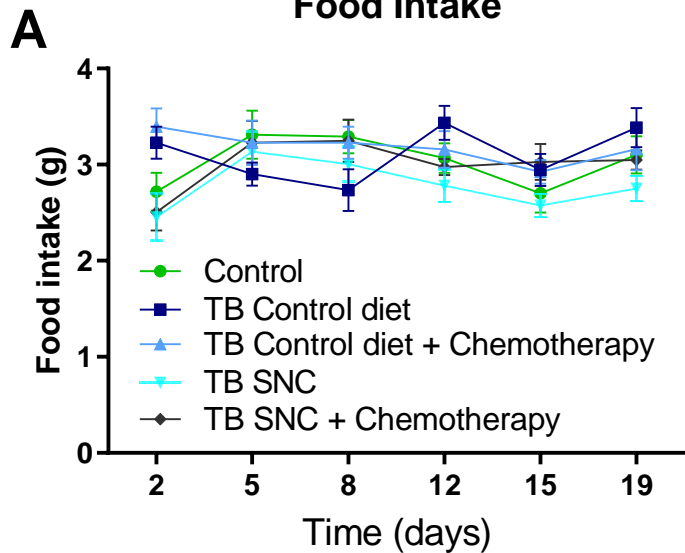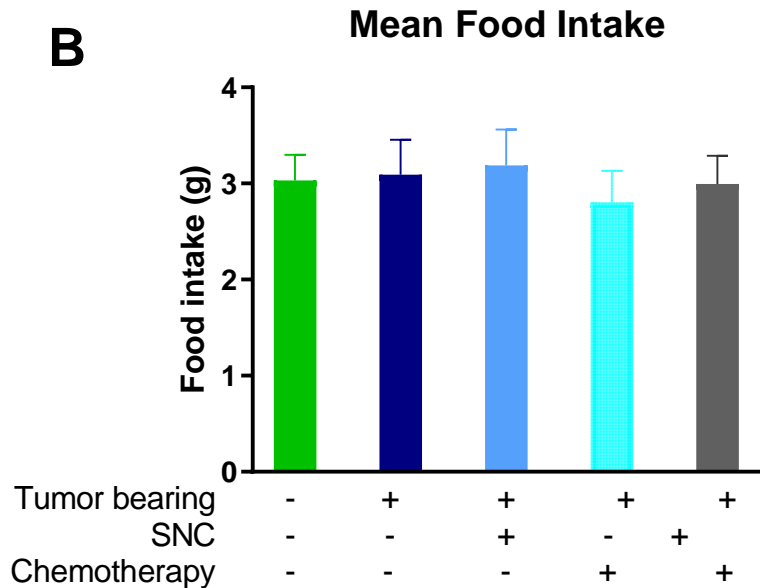

Supplement: Supplementary file 1 — Figure S1. Food intake. (A) Food intake per group at time points t = 2, 5, 8, 12, 15, 19. (B) Mean food intake per group [file JCSM-12-796-s005.pdf]

**A****Body weight**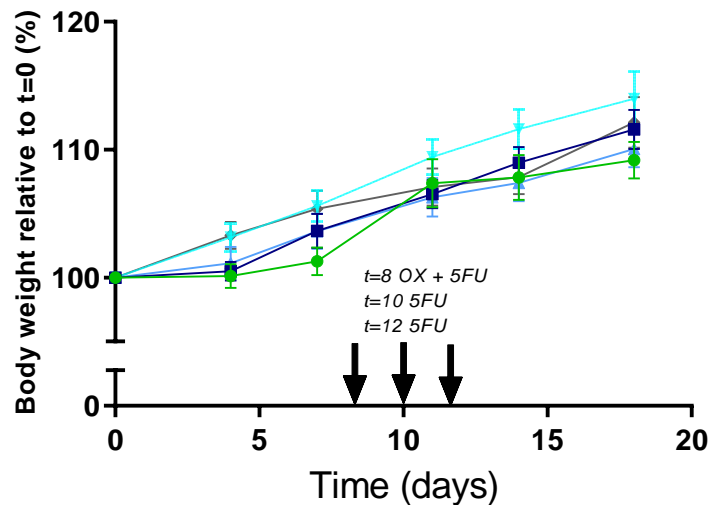

- Control
- TB Control diet
- ▲ TB Control diet + Chemotherapy
- ▼ TB SNC
- ◆ TB SNC + Chemotherapy

**B**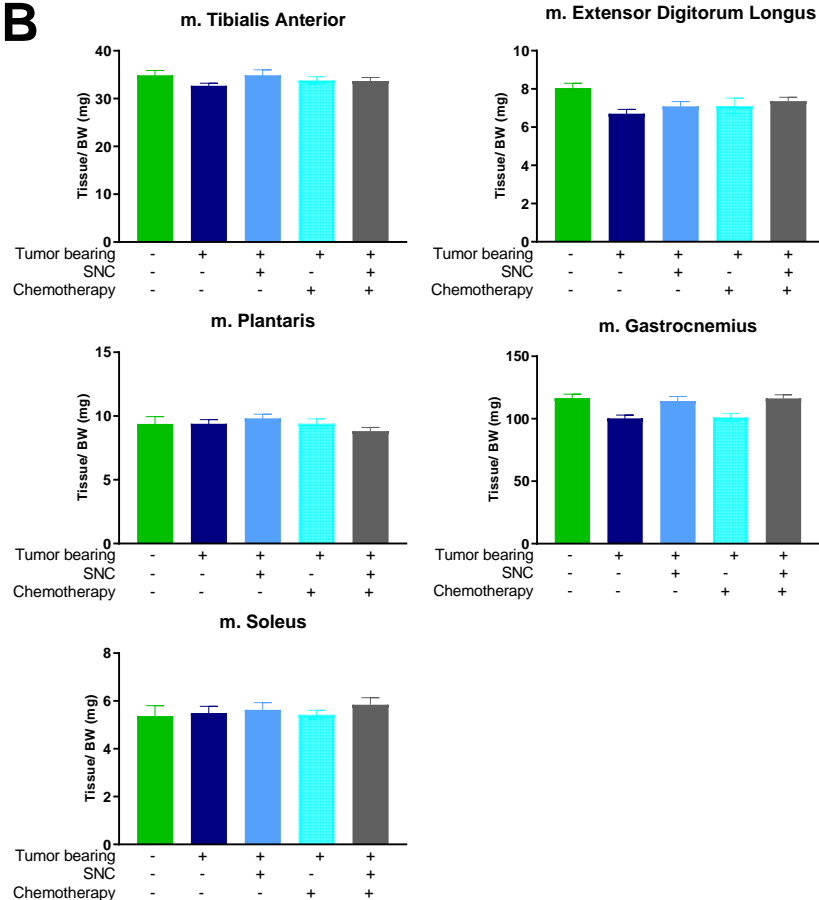

Supplement: Supplementary file 2 — Figure S2. Body weight and muscle weights. (A) Body weight (g), expressed as relative body weight to body weight at t = 0. (B) Muscle weights (mg) relative to body weight at sacrifice of m. Tibialis Anterior, Extensor Digitorum Longus, m. Plantaris, m. Gastrocnenius, m. Soleus [file JCSM-12-796-s002.pdf]

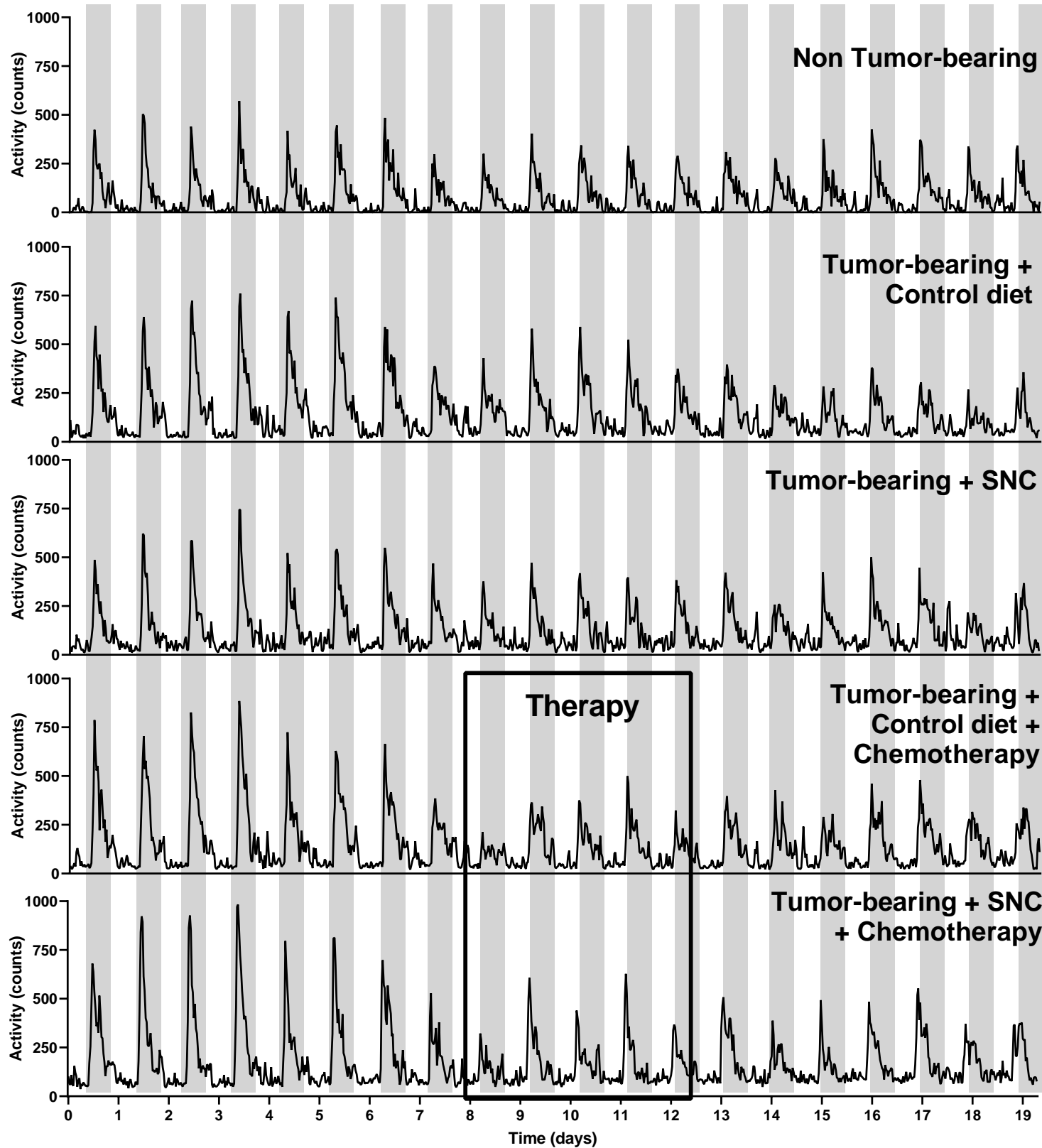

Supplement: Supplementary file 3 — Figure S3. Actogram of total physical activity over the course of the experiment. (A) Non‐TB mice show very little differences over time, whereas TB mice do show variances in physical activity. (B) During chemotherapy treatment (black box window) physical activity of mice fed with SNC is less impacted compared to control‐fed mice. Moreover, after chemotherapy mice fed with SNC show less disturbances in activity in the light phase compared to control‐fed mice. Non‐TB Control diet n = 8, TB Control diet n = 14, TB Control diet + chemotherapy n = 16, TB SNC n = 12, TB SNC + chemotherapy n = 15 [file JCSM-12-796-s004.pdf]
