# Supplementary material for: Specialized nutrition improves muscle function and physical activity without affecting chemotherapy efficacy in C26 tumour‐bearing mice
Source: J Cachexia Sarcopenia Muscle. 2021 May 6;12(3):796–810. doi: 10.1002/jcsm.12703 (PMC8200448; doi:10.1002/jcsm.12703)
Supplement: Supplementary file 4 — Data S1. Supporting Information [file JCSM-12-796-s003.docx]

# Specialized nutrition improves muscle function and physical activity without affecting chemotherapy efficacy in C26 tumour-bearing mice

Liza A. Wijler^1^, Danielle A.E. Raats^1^, Sjoerd G. Elias^2^, Francina J. Dijk^3^, Hanil Quirindongo^3^, Anne M. May^2^, Matthew J.W. Furber^3^, Bram Dorresteijn^3^, Miriam van Dijk^3^, Onno Kranenburg^1,4^

^1^ Laboratory of Translational Oncology, Division of Imaging and Cancer, University Medical Centre Utrecht, Utrecht University, Heidelberglaan 100, 3584CX, Utrecht, The Netherlands

^2^ Department of Epidemiology, Julius Center for Health Sciences and Primary Care, University Medical Center Utrecht, Utrecht University, P.O. Box 85500, 3508 GA Utrecht, The Netherlands

^3^ Danone Nutricia Research, Uppsalalaan 12, 3584 CT, Utrecht, the Netherlands

^4^ Utrecht Platform for Organoid Technology, Utrecht University. Heidelberglaan 8, 3584 CS Utrecht, The Netherlands

**Corresponding author:** Prof. dr. Onno Kranenburg, Laboratory of Translational Oncology, Division of Imaging and Cancer, University Medical Centre Utrecht, Heidelberglaan 100, 3584CX, Utrecht, The Netherlands. [o.kranenburg@umcutrecht.nl](mailto:o.kranenburg@umcutrecht.nl)

# Supplementary Methods

## Tumor organoid and cell culture medium specifications

CRC organoid culture medium contained Advanced Dulbecco’s modified Eagle medium (DMEM)/F12 (Invitrogen) with 1% Penicillin/Streptomycin (P/S) (Gibco), 1% HEPES buffer (Invitrogen) and 1% Glutamax (Invitrogen), 10% Noggin conditioned medium (produced by lentiviral transfection), 2% B27 supplement (Invitrogen), 1.25 mM n-Acetylcysteine (Sigma-Aldrich), 10 mM Nicotinamide (Sigma-Aldrich), 500 nM A83-01 (Tocris) and 10mM SB202190 (ApexBio).

## Basal Protein synthesis of C2C12 myotubes

After 48h incubation with SNCi and 24h treatment with chemotherapy (OXF), myotubes were deprived of L-leucine and serum for 4h. Newly synthesized peptides were labeled with 1 µM puromycin dihydrochloride (Calbiochem) for 30min and myotubes were fixated with 4% PFA (Merck) for 20min. Myotubes were permeabilized using 0.5% Triton X-100 (Sigma) for 5min and block buffer was added (PBS (Invitrogen) with 3% goat serum (Chemicon International) and 0.1% Triton X-100) for 1h. Myotubes were incubated overnight with anti-puromycin antibody (clone 12D10, 1:200, Millipore) in PBS with 3% goat serum and secondary antibody (Anti-mouse DyLight 488, 1:200, Molecular Probes) in PBS with 0.05% BSA (Sigma) for 1h. Fluorescence was measured using mounting medium AF1 (CitiFluor) using the Flexstation III Multi Mode plate reader (Molecular Devices); excitation 478nm and emission 518nm. Protein content was determined using the Amido Black Assay. Values are expressed as the ratio to control cells receiving only vehicle.

## Curve fitting of high throughput screening of patient-derived CRC organoids

A three separate four-parameter non-linear mixed-effect models - for each drug one - was used to fit dose-response curves in the presence or absence of SNCi, allowing fixed effect estimates of the top, bottom, and IC50 parameters to vary between SNCi and control, while estimating the slope as a single fixed effect across SNCi conditions (assuming that the average slope across organoids of the dose-response curve is not changed by SNCi). The formula of the four-parameter non-linear mixed-effect models was as follows:

Cell viability % = ((*top* - *bottom*)/(1 + exp(-(log[IC50] - log([chemotherapy])) / *slope* ))) + *bottom*

The fixed-effect bottom parameter estimates were constrained at 0% or higher viability, but were found to be zero for all models and were therefore excluded from the final analyses (i.e. bottom was fixed at zero). Five random effects were included to account for variability between organoid lines: for top and IC50 value parameters separately for the presence or absence of SNCi, in addition to a random slope per organoid line without accounting for SNCi. Random batch effects of the top parameter were included separately for each SNCi condition, nested within organoids. As the mixed-effect model for 5-fluorouracil would not converge with a random organoid slope, that parameter was omitted from that model. Differences in average IC50 and top viability estimates across organoids between the control and SNCi conditions were estimated from these non-linear mixed effect models and compared using Wald tests.
