# Supplementary material for: Specialized nutrition improves muscle function and physical activity without affecting chemotherapy efficacy in C26 tumour‐bearing mice
Source: J Cachexia Sarcopenia Muscle. 2021 May 6;12(3):796–810. doi: 10.1002/jcsm.12703 (PMC8200448; doi:10.1002/jcsm.12703)
Supplement: Supplementary file 5 — Table S1. Delta log IC50 and top viability values of tumor organoid killing curves with corresponding 95% confidence intervals (CI) and p‐values [file JCSM-12-796-s001.pdf]

Chemotherapy	log IC50		top viability (%)	
	Control diet	SNC	Control diet	SNC
Oxaliplatin	4.898	5.095	107.7	95.2
5-Fluorouracil	5.772	6.285	100.6	89.8
Irinotecan	3.449	3.715	99.9	90.4

Chemotherapy	delta log IC50	lower 95% confidence interval	upper 95% confidence interval	p-value
Oxaliplatin	0.197	-0.674	1.068	0.657
5-Fluorouracil	0.513	-0.093	1.118	0.097
Irinotecan	0.226	-0.544	0.997	0.564

Chemotherapy	delta top viability (%)	lower 95% confidence interval	upper 95% confidence interval	p-value
Oxaliplatin	-12.48	-31.21	6.26	0.192
5-Fluorouracil	-10.80	-41.72	20.12	0.493
Irinotecan	-9.47	-35.25	16.31	0.471
